# Systematic review of outcome measures in trials of pediatric anaphylaxis treatment

**DOI:** 10.1186/1710-1492-10-S1-A36

**Published:** 2014-03-03

**Authors:** Tamar Rubin, Jacqueline Clayton, Denise Adams, Hsing Jou, Sunita Vohra

**Affiliations:** 1 Department of Pediatrics, University of Alberta, Edmonton, Alberta, Canada; 2 CARE Program, University of Alberta, Edmonton, Alberta, Canada; 3 Department of Public Health Sciences, University of Alberta, Edmonton, Alberta, Canada

## Background

Considerable heterogeneity has been observed in the selection and reporting of disease-specific pediatric outcome measures in randomized controlled trials (RCTs) [1]. This makes interpretation of results and comparison across trials challenging [2]. Outcome measures in pediatric anaphylaxis trials have never previously been systematically assessed [3]. This systematic review (SR) will identify and assess outcome measures used in RCTs of anaphylaxis treatment in children. As a secondary objective, this SR will assess the evidence for current treatment modalities for anaphylaxis in the pediatric population.

## Methods

We searched MEDLINE, EMBASE, The Cochrane Library, Cochrane Central Register of Controlled Trials (CENTRAL), and CINAHL from 2001 until December 2012. We also searched websites listing ongoing trials. We included randomized and controlled trials of anaphylaxis treatment in patients 0-18 years of age. Two authors independently assessed articles for inclusion.

## Results

**Figure 1:**
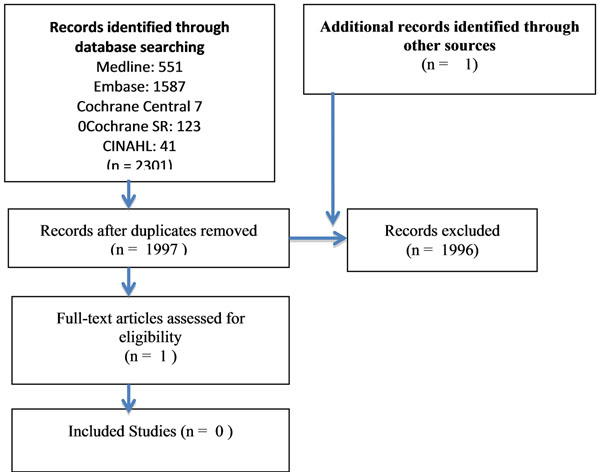
Search Flow Diagram

No published studies fulfilled the inclusion criteria (Fig 1).

## Conclusions

There is an alarming absence of RCTs evaluating the treatments for anaphylaxis in children. High quality studies are needed and are possible to design, despite the severe and acute nature of this condition. Consensus about the selection and validation of appropriate outcome measures will enhance the quality of research and improve the care of children with anaphylaxis.

## Systematic review registration

CRD42012002685
